# Workplace peer support for continuing professional development in occupational therapy: A grounded theory

**DOI:** 10.1111/1440-1630.70091

**Published:** 2026-05-05

**Authors:** Emmanuelle Moreau, Annie Rochette, Aliki Thomas, Marie‐Ève Caty, Brigitte Vachon

**Affiliations:** ^1^ École de réadaptation, Faculté de Médecine Université de Montréal Montréal Quebec Canada; ^2^ Centre de recherche interdisciplinaire en réadaptation du Montréal métropolitain Montréal Quebec Canada; ^3^ École de physiothérapie et d'ergothérapie, Faculté de Médecine et des Sciences de la santé Université McGill Montréal Quebec Canada; ^4^ Département d'orthophonie Université du Québec à Trois‐Rivières Trois‐Rivières Quebec Canada; ^5^ Institut universitaire en santé mentale de Montréal Centre de recherche du CIUSSS de l'Est de Montréal Montréal Quebec Canada

**Keywords:** learning, occupational therapists, peer support, professional knowledge transfer, reflection

## Abstract

**Introduction:**

Occupational therapists engage in continuing professional development (CPD) through various means, including workplace experiences. Workplace discussions between peers can promote reflection and foster learning. However, the specific contribution of peer support in occupational therapists' CPD remains underexplored. This study aimed to explore the role of peer support in clinical occupational therapists' CPD.

**Methods:**

A constructivist grounded theory approach was employed to develop an explanatory, substantive theory of the peer support process among occupational therapists working in clinical settings. Participants were occupational therapists who had received or provided peer support within the past 2 years. Semi‐structured interviews lasting approximately 60 minutes were conducted. Qualitative data were analysed using a constant comparative method involving initial, focussed, and theoretical coding.

**Consumer and Community Involvement:**

There is no consumer or community involvement.

**Findings:**

The 15 occupational therapists recruited had either only received (*n* = 4) or both offered and received (*n* = 11) peer support. Clinical reasoning, emotional support, professional growth, and career trajectories all acted as triggers for engaging in peer support. Analysis of participants' interview data informed the conceptualisation of peer support as an ongoing, non‐linear process shaped by three interrelated sets of conditions: (1) mobilisation (timely access, being grounded in practice, and access to relevant peer expertise), (2) interaction (navigating hierarchy, feeling safe, and dialogical conditions), and (3) practice context (workplace culture and protected time). Engagement in peer support was associated with a range of CPD‐related outcomes, including broadening perspective, embracing risk‐taking and practice‐based experimentation, building self‐efficacy, and transferring professional knowledge.

**Conclusion:**

The theory explains how the process of peer support contributes to multiple effects of CPD and fosters transformative learning at individual and organisational levels.

## PLAIN LANGUAGE SUMMARY

Occupational therapists continue learning throughout their careers to maintain their skills and provide high‐quality care. One way this learning happens is through everyday workplace experiences and conversations with colleagues. These peer discussions can support reflection and learning in practice. However, little is known about how peer support contributes to professional development. This study explored how peer support contributes to continuing professional development for occupational therapists working in clinical settings. Fifteen occupational therapists who had given or received peer support in the past 2 years took part in interviews lasting about 60 minutes. The occupational therapists shared their experiences of peer discussions at work. The findings showed that therapists sought peer support when they needed help with clinical reasoning, when facing emotionally challenging situations, or when reflecting on their professional development. How peer support unfolded depended on several conditions, including how easily peers could be accessed, whether discussions were based on similar work experiences, and whether peers had relevant expertise. It also depended on the quality of interactions—such as feeling safe, having open conversations, and managing differences in roles—as well as on the practice context, including workplace culture and protected time for discussion. Through peer support, therapists described gaining new perspectives on their practice, feeling more confident to try new approaches, strengthening professional confidence over time, and sharing knowledge with colleagues. Overall, this study highlights peer support as a process that continues over time and does not follow a single, predictable path, supporting learning in practice and contributing to continuing professional development at individual and team levels.

**Key Points for Occupational Therapy**

- Peer support was a relational process addressing clinical reasoning, emotional demands, and professional trajectories.
- Engagement in peer support was shaped by mobilisation conditions, interactional processes, and practice context.
- As an ongoing, non‐linear process, peer support contributed to CPD through multiple interconnected outcomes.

## 1 INTRODUCTION

Occupational therapists have a professional and ethical responsibility to maintain their competencies to ensure the quality of services provided and the protection of the public, notably through continuing professional development (CPD) (Alsop, 2013; Ordre des ergothérapeutes du Québec [OEQ], 2025a). CPD is commonly understood as a component of lifelong learning, supporting the development of professional expertise and the ongoing maintenance of competencies throughout practice (Alsop, 2013). CPD encompasses a broad range of formal and informal learning activities, whether intentional or incidental (Jarvis, 2024; Malcolm et al., 2003). Formal CPD activities typically involve structured education led by experts or facilitators and are often required and monitored by regulatory bodies (Jarvis, 2024). However, some of the learning that occurs throughout professional life is informal and incidental and embedded in everyday practice (Eraut, 2004), often taking place through interactions with colleagues (Jarvis, 2024). Learning in professional practice is frequently acquired through experience (Jarvis, 2024). When professionals encounter unexpected situations, a *disjuncture* arises between what is known and what is experienced, prompting reflection and further learning (Eraut, 2004; Jarvis, 2024). This process aligns with experiential learning theory, which conceptualises learning as a cyclical process involving concrete experience, reflective observation, abstract conceptualisation, and active experimentation (Kolb, 1984, 2015). From this perspective, everyday clinical situations and interactions with colleagues become key sites for CPD, transforming lived practice into opportunities for professional growth.

Importantly, workplace learning extends beyond purely cognitive processes. Illeris' (2002) three‐dimensional model of learning highlights the dynamic interplay between content (knowledge and skills), incentive (motivation and emotions), and interaction (social and organisational contexts). In professional settings, learning emerges through the integration of these dimensions, where motivation, emotional engagement, and social interaction shape how knowledge is constructed and applied. This study draws particularly on the interactional dimension of learning, emphasising how peer relationships, dialogue, and shared meaning‐making support professional development (Eraut, 2004; Illeris, 2002; Jarvis, 2024). Such an integrative perspective underscores that learning extends beyond individual processes to occur within social and organisational dynamics, favoured by collaborative activities such as observation, feedback, and peer support (Eraut, 2004; Stoll et al., 2006).

Peer support, through workplace discussion between peers, exemplifies this social dimension of learning by creating opportunities for collaborative reflection, feedback, and shared meaning‐making. In this study, ‘peers’ are understood as relational rather than hierarchical. ‘Peers’ refers to individuals who share professional interests, a common knowledge base, and mutual goals related to learning and practice improvement, regardless of formal role. Rather than constituting a single, clearly defined practice, peer support is variably described and may take multiple forms across professional contexts, ranging from informal and spontaneous exchanges to more structured arrangements (Alsop, 2013; Jarvis, 2024). This conceptual variability highlights the need to examine how peer support is understood and enacted by occupational therapists in everyday practice.

Research suggests that peer support is recognised for easing the transition into professional roles (Morley, 2006), particularly during early career stages when occupational therapists are developing confidence, learning new techniques, and seeking reassurance in their work with clients (Turpin et al., 2021). At all career stages, it fosters professional identity and resilience (Ashby et al., 2013), supports wellbeing and job satisfaction (Dupre & Salehi, 2025), and contributes to workplace retention (Ashby et al., 2013; Yan et al., 2024). It also serves as a complementary source of information in clinical decision‐making (Iqbal et al., 2023; Thomas, Roberge‐Dao, et al., 2023), as a source of reflection on practice (Rochette et al., 2020), and as a source of motivation and creativity (Dupre & Salehi, 2025).

Despite its recognised benefits, knowledge emerging from informal learning and peer‐based learning—often tacit and context sensitive—tends to receive limited recognition within dominant evidence‐based practice discourses (Eraut, 2004). In occupational therapy, tensions persist between research‐derived evidence and practice‐based knowledge, which may contribute to the latter being undervalued or insufficiently articulated in decision‐making (Greber & Isbel, 2024). Yet the complexity of practice contexts calls for a nuanced perspective, recognising that practice knowledge can complement scientific evidence when integrated into critical, contextualised clinical reasoning and professional judgement (Schell & Schell, 2024). In addition, perspectives on professional thinking highlight the role of *phronesis*, or practical wisdom, which develops over time through experience and reflective practice (Kinsella & Pitman, 2012). Practical wisdom draws on tacit or implicit forms of knowledge that are inherently difficult to articulate (Polanyi, 1966). Peer support is shaped by individual characteristics and varying engagement in evidence‐informed approaches; however, it should be viewed not as inconsistent with evidence‐based practice but as a complementary means of fostering growth and meeting practice demands when embedded in reflective and evidence‐informed professional dialogue.

This study aims to gain a deeper understanding of the underlying reasons why occupational therapists seek peer support for their professional development. Drawing on a transformative and experiential learning perspective, this study is guided by the following question: How does peer support contribute to meeting the professional needs of occupational therapists and support their learning and CPD?

## 2 METHODS

### 2.1 Study context

This study was conducted in the province of Quebec, one of Canada's 10 provinces. Occupational therapists are required to engage in CPD by their regulatory organisation, the OEQ, through a range of formal and informal learning activities (OEQ, 2025b). At the time of the study, participation in a minimum number of hours of formal CPD activities over a 3‐year cycle was required. However, participation in informal learning activities involving peers was recognised as engagement in CPD (OEQ, 2025b).

### 2.2 Design

Charmaz's (2014) constructivist grounded theory methodology was used, as it was designed to generate an explanatory theory grounded in participants' perspectives rather than test pre‐existing hypotheses. Constructivist grounded theory was well suited to explore processes related to seeking peer support and how workplace interactions shape learning experiences. Its constructivist orientation acknowledged that knowledge is co‐constructed between researchers and participants, allowing reflexivity and contextual understanding to inform analysis (Charmaz & Belgrave, 2019). The COREQ (Consolidated Criteria for Reporting Qualitative Research) checklist was used to ensure methodological quality. Ethical approval was obtained from the university's research ethics board (2021‐1113, CERSES‐21‐083‐D). This study adhered to the World Health Organization research ethics standards with human participants (Health Ethics & Governance, 2011). All participants provided informed consent before study involvement.

### 2.3 Positionality statement and reflexivity

The research team comprised five members with professional backgrounds in occupational therapy and speech‐language pathology, all with experience in qualitative research. The project lead, an occupational therapist with experience in both the public and private sectors, had previously received and provided peer support. Grounded in a constructivist paradigm (Charmaz, 2014), this research team sought to understand peer support from the occupational therapists' perspectives. Consistent with Kolb (1984, 2015) and Illeris' (2002) framework, they shared the assumption that learning mobilises three dimensions—cognitive (content), emotional (incentive or motivation), and social (interaction)—and that workplace experience can be understood as part of a CPD journey. The project lead's professional backgrounds and prior engagement with peer support may have shaped an initial broad conceptualisation of the concept, leading them to search for the positive experiences peer support had on practice. To manage these assumptions, a reflexive stance was adopted throughout the study, including memo writing and ongoing analytic discussions within the research team.

### 2.4 Procedure

#### 2.4.1 Recruitment and sampling

Occupational therapists working in the province of Quebec were recruited using an initial non‐probability convenience sampling strategy, complemented by snowball sampling (Jacques et al., 2020). Inclusion criteria were (a) registration with the provincial regulatory body, (b) having received or provided peer support in the past 2 years, and (c) sufficient fluency in French.

Participants were recruited through professional networks and social media platforms targeting occupational therapists. Eligible participants completed an online screening form and received study information and consent materials before scheduling an online interview. All interviews were conducted in French and analysed in their original language. Selected excerpts were translated into English using DeepL software (2024) and reviewed by two bilingual individuals to ensure semantic accuracy (Tarozzi, 2019).

As data collection and analysis progressed, theoretical sampling was used to guide ongoing recruitment. Early analyses highlighted variations in access to and engagement in peer support across practice contexts, particularly between public and private practices, as well as differences related to professional experience. These insights informed the purposeful recruitment of participants with diverse practice contexts and varying levels of experience. Additional participants were purposefully recruited through targeted professional networks and social media platforms to include occupational therapists working in private practice and recent graduates.

#### 2.4.2 Data collection

Data were collected between June 2023 and January 2024 by the first author. Consistent with the constructivist grounded theory, data collection and analysis occurred concurrently, allowing iteratively developed analytic insights to inform subsequent interviews and guide theoretical sampling decisions (Charmaz, 2014). Semi‐structured interviews were conducted to explore participants' experiences of peer support (Charmaz & Belgrave, 2021). An interview guide was developed, following the steps outlined by Kallio and collaborators (2016), reviewed by an occupational therapist with expertise in mentorship (a non‐member of the research team), and pre‐tested with two clinical occupational therapists who were engaged in direct client care (see Table 1). As interviews progressed, minor adjustments were made to the wording and sequencing of questions to reflect the language and concepts raised by participants and to explore areas of interest constructed through participants' accounts and ongoing analysis (Charmaz, 2014). Participants completed a socio‐demographic data form prior to the interview (see supporting information). Interviews were conducted using Microsoft Teams, whose built‐in transcription tool automatically converted the recordings into text. A research assistant subsequently reviewed the transcripts for accuracy and anonymity. The first author documented their ideas and reflections in memos (Charmaz, 2014).

**TABLE 1:** Semi‐structured interview guide questions and specific probes.

Questions	Specific probes
Can you describe your current practice? For example, how long have you been practising, what are the characteristics of your work environment, what are your clients' characteristics, and any other information about your context that you think I should know?	
Can you explain what peer support means to you?	
In what forms do you use or provide peer support at work?	How often do you use peer support? What form do you think responds best to your own or the other person's needs? Do you have examples?
Can you think of a positive, significant moment of peer support?	What motivated you (or the other person) to seek peer support in this situation? Based on your experience, what do you believe you have learned through the peer support process (knowledge, skills, and attitudes)? Based on your experience, how did you feel before, during, and after receiving or providing support?
More broadly, how does peer support influence your motivation to engage or not in continuing professional development?	
How does peer support influence social aspects of your practice such as your professional identity, your place within your team, and your role within your organisation?	
Now that we have discussed a positive example, do you have any experience of a peer support moment that was less positive or that did not work out for you?	
Now that you have described your previous peer support experiences, what would be an ideal experience? How would peer support happen in an ideal world?	What are the specific characteristics of the people involved? What happens in the support moment itself? What are the environmental characteristics (either social, structural, or physical)?
If you think peer support, as you described it, should be promoted, how should we promote it?	
Is there anything else we have not covered that you would like to share?	
Do you have any final words? What would you want me to take away from today's discussion? Are there any ideas that you want us to remember for this study?	

#### 2.4.3 Data analysis

Data were managed using NVivo 14 software (Lumivero, 2023). Analysis followed Charmaz's (2014) grounded theory procedures, including initial, focussed, and theoretical coding, supported by constant comparison of data and memo writing. Initially, line‐by‐line coding was carried out to segment the transcript content and uncover subtle distinctions in the occupational therapists' words (e.g., type of peer support and outcomes). A comparison of incidents (e.g., clinical situations and peer support significant moments) enabled us to discern the events that led occupational therapists to solicit their peers. During the coding process, new ideas were compared with existing codes to identify contradictions and to refine the initial codes. During the focussed coding process, the initial codes were compared to each other to determine their relative importance, expand their meaning, and restructure them into broader categories. Next, theoretical coding was used to organise the categories more clearly and to explore the links between them. This constant comparison process, supported by ongoing memoing, also allowed for the refinement of categories, the creation of new ones, and the grouping of categories. Theoretical sampling was used as an analytic strategy to refine emerging categories. Early analyses raised questions that guided additional data collection to further develop category properties and relationships. This iterative process supported the integration of categories within the developing theoretical framework. Data collection and analysis continued until theoretical sufficiency was reached, with no new conceptual insights from the data (Charmaz, 2014).

### 2.5 Quality and trustworthiness of the study

To enhance credibility, the research team maintained close engagement with the data through repeated checking of transcripts, memo writing, constant comparison across interviews and analytic categories, and ongoing analytic discussions (Charmaz, 2014). Coding and interpretations were reviewed collaboratively at multiple stages of analysis. Finally, conducting the analysis and manuscript preparation in French helped preserve proximity with participants' meanings and experiences.

## 3 FINDINGS

### 3.1 Participants' characteristics

Fifteen occupational therapists, working in multiple practice settings in Quebec, participated in the study (see Table 2). Fourteen were identified as women and one as a man (mean age of 32 years, ranging from 24 to 48 years). Participants had an average of 8 years of professional experience and 4.5 years in their current position. Eleven both received and provided peer support, whereas four reported only receiving support.

**TABLE 2:** Participants' practice, team characteristics, and peer support available.

ID	Practice context	Population	Experience as an OT (years)	Experience at this workplace (years)	Team size	Number of OTs in the team	Received or offered peer support	Type of support available (questionnaire)	Type of support used (interview)
E01	RC, PP	Adults	4	4	6–10	6 or more	Both	Community of practice (CoP), scheduled discussion with a colleague	Mentorship, research collaboration, sharing and discussion
E02	RC, PP	Infants, youths	2	2	6–20	6 or more	Both	Coaching, co‐development (CD), CoP, scheduled and unscheduled discussion with a colleague, mentorship, clinical supervision	Coaching, **mentorship**, sharing and discussion
E03	RC	Adults	4	<1	6–10	2–3	Received	CoP, scheduled and unscheduled discussion with a colleague, mentorship, sponsorship, clinical supervision	**Sponsorship**, **sharing and discussion**, clinico‐administrative support
E04	RC	Infants	1	1	11–15	4–5	Received	Coaching, CD, CoP, scheduled and unscheduled discussion with a colleague, mentorship, sponsorship, clinico‐administrative supervision, clinical supervision	**Sponsorship**, sharing and discussion, **clinico‐administrative support**, group discussion
E05	PP	Adults	<1	<1	6–10	Alone for the first 6 months. Then another OT joined the team	Received	Mentorship, other	Mentorship, sharing and discussion, group discussion, social media network
E06	PP	Youths	10	5	1–5	Solo OT	Both	Unscheduled discussion with a colleague, other	Coaching, sharing and discussion, research collaboration, social media network
E07	LTCF	Geriatrics	10	10	6–10	Solo OT	Both	CoP, unscheduled discussion with a colleague, mentorship	Sharing and discussion, fieldwork supervision, community of practice
E08	PMATS	Adults, geriatrics	2	2	16–20	6 or more	Both	Coaching, scheduled and unscheduled discussion with a colleague, sponsorship, clinical supervision	**Sponsorship**, sharing and discussion, clinico‐administrative support
E09	PP	Youths, adults, geriatrics	19	3	11–15	2–3	Both	Unscheduled discussion with a colleague, mentorship	Mentorship, sharing and discussion, fieldwork supervision
E10	PMATS	Adults, geriatrics	7	3	40	6 or more	Both	Coaching, CD, scheduled and unscheduled discussion with a colleague, mentorship, sponsorship, clinico‐administrative supervision, clinical supervision	**Sponsorship**, sharing and discussion, fieldwork supervision, group discussion
E11	LTCF	Geriatrics	24	10	1–5	Solo OT	Both	CoP, scheduled and unscheduled discussion with a colleague	Sharing and discussion, community of practice, group discussion
E12	PP	Adults	8	1	1–5	Solo OT	Both	Scheduled and unscheduled discussion with a colleague, mentorship, sponsorship	Mentorship, sharing and discussion
E13	PMATS	Adults, geriatrics	19	16	16–20	6 or more	Both	Scheduled and unscheduled discussion with a colleague, sponsorship, clinico‐administrative supervision, clinical supervision	Sponsorship, sharing and discussion, clinico‐administrative support, group discussion
E14	PCS	Adults, geriatrics	5	5	16–20	6 or more	Received	Scheduled and unscheduled discussion with a colleague, mentorship	Mentorship, sharing and discussion
E15	PMATS	Adults	6	6	16–20	6 or more	Both	Scheduled and unscheduled discussion with a colleague, mentorship, clinical supervision	Sponsorship, sharing and discussion, clinico‐administrative support, fieldwork supervision, group discussion

*Note*: In bold indicates orientation and introduction to a new workplace.

Abbreviations: LTCF, long‐term care facility; OTs, occupational therapists; PCS, primary care services; PMATS, Posture and Mobility Assistive Technology Services; PP, private practice; RC, rehabilitation centre.

### 3.2 Peer support definition as perceived by participants

In the absence of a consensual definition in the literature, participants were invited to describe peer support in their own words (see Table 3). Across accounts, peer support was described as a relational and reciprocal process grounded in shared practice realities, characterised by egalitarian and non‐evaluative exchanges through which colleagues provided clinical, reflective, and emotional support.

**TABLE 3:** Core features of peer support as described by participants.

Core features	Key excerpts
Relational process of mutual exchange	A concept of knowledge sharing, sharing of expertise where you give and receive (E02) It can take the form of exchanging knowledge, documents … I also receive information (E09) Both when we give and when we receive (E12)
Availability	Someone who will be available to answer your questions (E01) Availability … time, a presence (E07) What matters most … is the attitude […] having someone who genuinely wants to help you (E14)
Relatively egalitarian and non‐evaluative relationship	It's different from mentoring … there is a form of equality (E05) No authority, no ‘I'm evaluating you’ (E06) I have always learned with my interns … I consider them at the same level as me. [However,] there is a kind of hierarchy … because the student still feels in an evaluative context (E09)
Sharing common professional realities and practice‐related concerns	My colleagues who are occupational therapists (E03) It's really interdisciplinary (E14) A peer can also be … another health professional (E06)
Clinical, reflective, and emotional support	Supporting the development of my professional practice, the development of my competencies (E03) Brainstorming … making sure your ideas make sense in clinical and ethical contexts (E05) Emotional support, between peers who understand your realities (E05)

### 3.3 Role of peer support in occupational therapy

**FIGURE 1:**
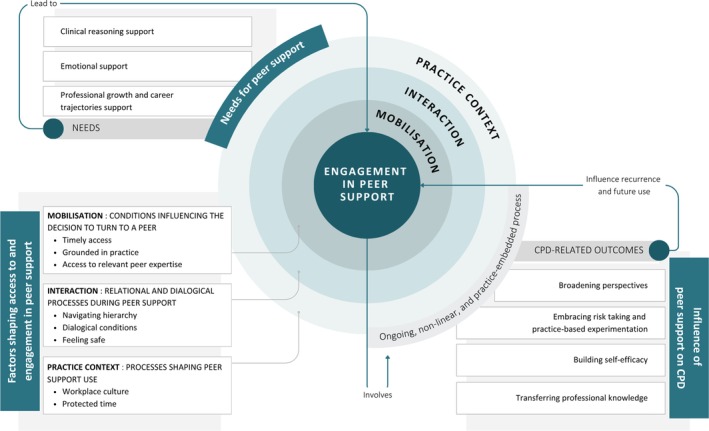
Explanatory substantive theory of the process of peer support among occupational therapists working in clinical settings.

Peer support in occupational therapy was conceptualised by participants as an overarching, non‐linear, and practice‐embedded process (Figure 1), triggered by different needs for peer support, influenced by access to and engagement in peer support, leading to multiple overlapping CPD‐related outcomes that influenced future use of peer support.

#### 3.3.1 Needs for peer support

The main reasons for seeking peer support were (1) clinical reasoning support, (2) emotional support, or (3) professional growth and career trajectories support.

##### Clinical reasoning support

Regardless of career stage, occupational therapists sought peer support to validate clinical reasoning and to gain an alternative perspective. This was particularly salient in complex or unfamiliar situations requiring tailored decision‐making. As one participant explained, peer support helped ‘to verify a decision, to ask for advice on the choice of a practical simulation, or to validate assessment results’ (E03). Another participant would ask a peer: ‘Do you have any other advice? Has this ever happened to you in the past?’ (E04) to initiate exchanges and benefit from a colleague's experience. These exchanges also reflected elements of conditional reasoning, as practitioners drew on both their own and their peers' experiential knowledge to anticipate how different decisions might unfold in specific client contexts.

##### Emotional support

Participants also sought peer support to manage emotionally demanding situations. For instance, one occupational therapist spoke of ‘[their] emotions, [which they] saw as bubbling up inside [them]’ (E08), which could prompt them to seek peer support. Although not directly related to skill acquisition, emotional support enabled therapists to gain distance and reframe challenging experiences, thereby supporting continued engagement in practice, as explained by one occupational therapist: ‘having talked to him gave me much more perspective on a negative experience’ (E05). Another said they offered different kinds of support to their colleagues: ‘whether it is work‐related information or helping to ventilate. It's a bit more psychological in that regard’ (E10). By helping therapists manage emotional strain, peer support created conditions that sustained engagement in practice and learning.

##### Professional growth and career trajectories support

Peer support for professional growth was typically mobilised on a more planned and intentional basis to support skill development and longer term professional trajectories. One participant described how, when an occupational therapist wished to develop a specific area of expertise, ‘there's a kind of agreement that's made […] [and then, the manager] unblocks an advanced mentorship programme’ (E02), referring to a clinic‐based arrangement in which an occupational therapist with advanced expertise provided targeted peer support and dedicated exchange time supported by the employer. Such structured forms of peer support were described as more commonly embedded within public sector settings, particularly through formal accompaniment of mentorship structures at entry into practice. As one participant who had worked in both sectors explained:

In the public sector, there is a structure in place […] if you don't have your equivalent, there is someone you can refer to […] resources and tools are provided by the employer […] which is really not the case in private practice. (E12)

Overall, these accounts illustrate how peer support for professional growth and career development was more formally organised and institutionally supported in public sector contexts, whereas in private practice, it tended to remain more ad hoc and individually negotiated.

#### 3.3.2 Factors shaping access to and engagement in peer support

The decision to consult a fellow professional rather than another source of information was influenced by processes related to peer support mobilisation, interaction conditions, and features of the practice context (see Figure 1).

##### Mobilisation: Conditions influencing the decision to turn to a peer

Participants described mobilising peer support because it was timely, grounded in practice, and enabled access to relevant expertise.

###### Timely access

Peer support was valued as an immediate and flexible strategy that could be mobilised in the flow of practice, often in response to emerging questions or emotional strain. For example, one participant said, ‘We're working. Then it *pops* into my head, and I feel like talking about it’ (E02). This immediacy facilitated help‐seeking when therapists were ‘in the thick of it’ (E13) and needed rapid clarification or reassurance. Timely access was therefore a key condition shaping the initial decision to turn to a peer rather than consult more formal or delayed sources.

###### Grounded in practice

Peer support was also mobilised because it was embedded in shared clinical realities, making exchanges concrete and directly applicable. An occupational therapist mentioned seeking support: ‘[…] [from] someone […] who can provide examples of similar clients [because] it makes it easier to understand with case histories’ (E15). Participants emphasised that peers could draw on comparable cases and anticipate practice‐specific challenges, supporting reflection and clinical reasoning in ways that abstract sources could not. Specifically, ‘the occupational therapist who's going to help me in a situation will ask me the right questions, they will proactively anticipate the challenges I will face. Whereas a book can't necessarily do this’ (E14). This practice‐grounded quality recurred across participants' narratives.

###### Access to relevant peer expertise

Occupational therapists selectively mobilised peers based on perceived expertise, experience, or interest in a given area. ‘Choosing the right person’ (E10) enabled therapists to ‘really go out and find the person according to the expertise we're looking for’ (E03). Another participant explained from the perspective of providing peer support: ‘We all have the same basic knowledge, but with our experience, and our interest too, there are subjects in which we feel more able to provide support’ (E07). Furthermore, some occupational therapists also valued the complementarity of experiential knowledge from colleagues in other disciplines, such as physiotherapy (E01 and E09), or from a mechanical technician (E15). This recognition of differentiated expertise contributed to the purposeful and targeted mobilisation of peer support.

##### Interaction: Relational and dialogical processes during peer support

Participants highlighted that relational and dialogical processes, including navigating hierarchy, dialogical conditions, and feeling safe, shaped the quality and usefulness of peer support interactions across their experiences.

###### Navigating hierarchy

Hierarchy was experienced in two forms: experiential hierarchy (linked to clinical experience) and positional hierarchy (linked to organisational roles). Regarding clinical experience, some participants sought it, seeing it as ‘a source of aspiration to become like that’ (E02). One occupational therapist mitigated this tendency: ‘[…] we tend to constantly call on the older person, whereas sometimes, the younger ones surely have a lot to teach us too’ (E03). As for organisational hierarchy, it could be sought or avoided, depending on the occupational therapist's needs. For instance, a participant stated that they would consult their professional coordinator when situations were more precarious, ‘to protect themselves’ (E15). In contrast, another occupational therapist characterised peer support as a ‘safe space’ (E05) that they did not find with someone in a position of authority: ‘I wouldn't mention out loud, you know, if I had doubts or things like that’ (E05).

###### Dialogical conditions

Participants valued dialogical exchanges characterised by openness, flexibility, and reflective questioning. Reflective approaches were preferred and described as ‘discussing, deepening your thoughts, structuring your clinical reasoning in relation to specific issues, and then helping this initial process towards identifying areas of development’ (E02). It also involved ‘learning to dig around for alternatives, […] really to push your thinking to come up with new ideas’ (E13). In contrast, directive interactions were sometimes experienced as limiting reflection and undermining confidence, as stated by this participant when asking for support from a peer, who responded by saying: ‘“This is what you have to do: this, this, this, this, this, this.” For me, it doesn't echo. Then I realised that sometimes, I really didn't feel competent […]’ (E02). Furthermore, conversations rooted in inflexible viewpoints, or ‘absolutes’ (E12), were considered ‘detrimental to creativity or professional independence […] since it was not allowing to come up with one's own solutions’ (E03). Such dialogical conditions were described as limiting opportunities for autonomous reflection and constraining the development of professional judgement.

###### Feeling safe

Feeling safe emerged as a prerequisite for meaningful peer support. Psychological safety enabled participants to ‘put [themselves] in a vulnerable position’ (E10) and to ‘dive into [their] vulnerability and then say, “I did that, I'm not sure”’ (E06). A dynamic of ‘non‐judgment [… and] feeling that it's received with kindness when we ask questions’ (E05) was identified by another participant as a necessary component, ‘to dare to continue asking questions’ (E05). In its absence, participants described hesitating to seek support or moderating what they disclosed.

##### Practice context: Processes shaping peer support use

The practice context influenced access to and engagement in peer support through workplace culture and protected time.

###### Workplace culture

Supportive cultures normalised help‐seeking and made peers' availability visible, thereby facilitating informal exchanges. An occupational therapist said: ‘The person […] has to know that there are people around her, that this is known, that there are people who can help. After that, it's up to them to ask for help’ (E11). Even when the availability of support was known, the messages conveyed by team members influenced whether or not support was sought. A participant said that hearing ‘sentences such as “don't forget that my door is always open” [made them feel that they] could go and see them at any time’ (E03). In contrast, productivity pressure—particularly in private practice—often constrained opportunities for peer dialogue and positioned peer support as discretionary rather than valued aspects of work. One participant captured this tension by stating that peer support felt like ‘doing volunteer work’ (E02). Another noted that exchanges between colleagues were ‘always [taking] place between two clients, quickly quickly’ (E09), limiting opportunities for meaningful discussions. Participants also expressed a desire for a workplace culture that legitimises peer support, where ‘it's okay to call your colleague and ask for help, even if this does not fit into the statistics’ (E06).

###### Protected time

Although immediate exchanges met short‐term needs, participants emphasised that protected time was essential for deeper dialogue and reflection. ‘[T]aking the time’ (E08) and ‘not feeling rushed’ (E01) were deemed as ‘necessary to encourage active exchanges [between those involved]’ (E01) and enable ‘richer reflections’ (E02). Time constraints limited the depth of discussion, whereas dedicated time enabled sustained engagement and more meaningful exchanges that could lead to CPD opportunities.

#### 3.3.3 Influence of peer support on CPD

The influence of peer support on CPD was described through a set of interrelated outcomes that supported learning as an ongoing, non‐linear, and practice‐embedded process (see Figure 1). These outcomes included broadening perspectives, embracing risk‐taking and practice‐based experimentation, building self‐efficacy, and transferring professional knowledge. Although presented separately for clarity, participants' accounts suggested that these outcomes were closely intertwined and mutually reinforcing in practice.

##### Broadening perspectives

Peer discussions enabled occupational therapists to step back from habitual ways of thinking, examine underlying assumptions, and consider alternative viewpoints in clinical situations. Participants emphasised the value of having another professional perspective that allowed for disagreement and critical dialogue: ‘[it] is important for me, to have another perspective […] to be able to […] say if I disagree or give my own opinion’ (E08). Such exchanges supported reflective processes through which therapists described revisiting ‘our ways of doing things, and our a priori’ (E12). These reflective processes sometimes led to an initial awareness of previously unexamined assumptions, as illustrated by statements such as ‘we don't know what we don't know’ (E02) and ‘after that, I'm in another set of minds’ (E10). For others, the broadening of perspectives was described as an ongoing process rather than a discrete moment of change: ‘it's rare that the process leads to it happening, and then it's over. For me, it's more of an ongoing thing’ (E12).

##### Embracing risk‐taking and practice‐based experimentation

Peer support also fostered confidence to trial new approaches to assessment and intervention in practice. Participants described peer reassurance as legitimising action and reducing uncertainty, particularly when interventions aligned with what experienced colleagues would do: ‘OK, I can go ahead. It's the same thing that a very experienced colleague would do. So, my plan is good’ (E14). Beyond reassurance, peer exchanges supported experimentation that referred to testing practice changes in real clinical contexts, supported by peer dialogue. These discussions allowed occupational therapists to explore the rationale underpinning interventions, develop practical know‐how, and plan their integration into practice:

For me, it's with peer support that we can optimise the integration of new tools into the workplace environment by integrating it and then saying to others: ‘Well, have you tried it? And how do you do it? And how do you think about it?’ (E01)

However, motivation to experiment depended on how support was provided; directive advice without shared understanding limited uptake, whereas ‘co‐constructing a solution together’ (E03) enhanced willingness to try new approches in practice.

##### Building self‐efficacy

Positive peer support experiences strengthened therapists' confidence in managing future situations. Validation from peers reinforced clinical judgement and supported a sense of professional solidarity, particularly following emotionally challenging cases: ‘Then it seems like I'd be more solid if a similar situation arose […]. [I]t's not just me who's thinking like this […] my colleague also thinks like this’ (E05). Over time, these experiences contributed to a growing sense of self‐efficacy, enabling therapists to approach similar situations with greater confidence and autonomy: ‘[I]t really helped me, because I had other cases, afterwards. […] I used this [previous] meeting with my colleague […] to start from an evaluation plan that I felt confident with’ (E07).

##### Transferring professional knowledge

Receiving peer support often generated a desire to reciprocate and share knowledge with others, contributing to collective learning. Participants described a motivation ‘to give back to the next person’ (E04), to ‘pass on information [to] the future generations’ (E13), and to sustain expertise within teams, particularly in contexts marked by staff turnover:

We kind of lost all our expertise at the same time. So, there's something about valuing the descent of expertise, [as a] cascade of knowledge. […] [Y]ou have to value clinical experience [… and] informal discussions [because,] in the end, it has also impacted the organisational level—this loss of knowledge, the loss of expertise. (E02)

However, some pointed out the disadvantage of relying solely on peer‐to‐peer knowledge validated only through experiences: ‘I find that this is what sometimes contributes to making the environment a little sterile, too much peer support’ (E03).

## 4 DISCUSSION

This study explored how occupational therapists from diverse practice domains experienced the role of peer support as a component of their CPD. The substantive explanatory theory developed positions peer support as an ongoing, non‐linear, and practice‐embedded process shaped by interrelated conditions related to the mobilisation of peer support, interaction, and practice context (Figure 1). Through peer support, occupational therapists transformed lived professional experiences into learning by fostering reflection and clinical reasoning, addressing emotional support needs, and supporting professional growth and career trajectories.

### 4.1 Role of peer support

Grounded in participants' accounts, peer support was understood as a relational and reciprocal process, rather than a role defined by formal position or hierarchy. Peers were identified through shared practice realities and exchanges experienced as egalitarian, non‐evaluative, and oriented towards mutual learning.

Peer support in occupational therapy was mobilised primarily for clinical reasoning, emotional support, professional growth, and career trajectories. In relation to *clinical reasoning*, peer exchanges created opportunities for reflection, joint problem‐solving, and the articulation of tacit knowledge, resonating with Schön's (1983) notion of reflection‐on‐action. Rather than relying on abstract recommendations derived from external knowledge sources, participants described actively seeking peer input to test the feasibility of clinical options in relation to contextual constraints such as time, organisational expectations, clients' needs, or available resources. These exchanges supported pragmatic and conditional reasoning grounded in the realities of practice (Schell & Schell, 2024) and facilitated the emergence of socially situated knowledge embedded in workplace culture (Eraut, 2004; Schell & Schell, 2024). These processes may also contribute to the development of practical wisdom, which Kinsella and Pitman (2012) note is difficult to teach directly. Through dialogue with peers, occupational therapists described examining, questioning, and reworking their clinical reasoning and decisions, which strengthened their ability to respond to complex practice contexts and informed future actions. As illustrated in Figure 1, such dialogical exchanges were actively sought as a means of supporting clinical reasoning, a finding that is consistent with previous research showing that peer support can facilitate reasoning, critical thinking, and decision‐making (Jackson et al., 2023; Robertson, 2012).

A prominent dimension of peer support concerned *emotional support*. Debriefing with peers after challenging cases or when facing self‐doubt could help to regulate emotions and reduce stress (Jackson et al., 2023). In that sense, emotional reassurance primarily fostered psychological safety (Edmondson, 1999). Consistent with Illeris' (2002) learning model, supporting the emotional dimension appeared to create conditions that freed cognitive resources for reflection and problem‐solving. Such processes resonate with approaches emphasising mindfulness and self‐care, which may support attention, self‐awareness, and non‐judgement and may contribute to professional development by enhancing presence and emotional regulation (Kinsella et al., 2020). Beyond immediate emotional benefits, peer‐based emotional support also functioned as a key pillar of professional resilience, enabling occupational therapists to sustain their professional identity, develop adaptive coping strategies, and support career longevity in the face of institutional pressures (Ashby et al., 2025). However, emotional support was not always conducive to learning. As reported in studies on reflective practice (Mann et al., 2009) and workplace learning (Eraut, 2004), some participants described exchanges that mainly served as venting or receiving comfort, helping them regain emotional balance without leading to practice change. Furthermore, excessive emotional support without critical questioning may confine professionals to a comfort zone that reinforces existing practices, whereas deeper professional development requires moving beyond reassurance towards reflective and critically oriented dialogue (Øen et al., 2024). This emphasises the dual nature of peer support. Although emotional support is not typically conceptualised as CPD in itself, when embedded in reflective, critical, and dialogical exchanges, it may create conditions conducive to CPD‐related outcomes (Lindblom et al., 2025). In this sense, connecting debriefing to further dialogue can transform emotional experiences into springboards for professional growth (Evans et al., 2023).

A third form of engagement in peer support concerned *professional growth and career trajectories*. Participants described connecting with like‐minded colleagues with recognised clinical expertise to support both practice‐based skill development, reflection on longer term professional pathways, and engagement in their CPD. Peer credibility and expertise were central to these exchanges, echoing findings that occupational therapists identify role models and mentors who motivate engagement with best practices (Hallé et al., 2018). Experiential hierarchy also shaped these exchanges, with occupational therapists deliberately approaching experienced peers able to support both advanced clinical development and access to wider professional networks, thus fostering career opportunities and long‐term growth (Kuhaneck, 2010). Such intentional use of peer support aligns with evidence that peers and mentors can shape professional trajectories in health professions (Mann et al., 2009).

Overall, although peer support is not designed to replace formal continuing education or systematic engagement with research evidence, these findings suggest that it plays a distinct and essential role by supporting emotional, social, and situated dimensions of learning that are less easily addressed through formal, cognitively oriented CPD activities. Peer support thus emerged as a complementary strategy that sustains learning embedded in everyday practice.

### 4.2 Importance of peer choice, interaction, and practice context

Consistent with the explanatory theory presented in Figure 1, engagement in peer support was shaped by three interrelated levels: mobilisation processes related to the support itself, relational and dialogical interactional conditions, and features of the practice context.

Peer support *mobilisation* processes were central to decisions to engage in peer support. Occupational therapists mobilised peer support when it offered timely access, was grounded in shared practice realities, and provided access to relevant peer expertise. *Timely access* facilitated reassurance or rapid access to information when practitioners were ‘in the thick of it’. However, immediacy did not guarantee the protected time required for deeper reflection, which participants and other studies viewed as essential for more complex learning processes (Marshall et al., 2022). Peers were prioritised over other sources of information because their experiential and practice‐based knowledge was immediately applicable to local clinical situations, saving time and avoiding more abstract forms of reasoning (Rochette et al., 2020). This *grounding in shared practice realities* enabled peer support to oscillate between offering concrete, situation‐specific solutions and facilitating the co‐construction of mindlines—tacit, practice‐grounded, and collectively reinforced ways of knowing shaped by experience, professional exchange, and clinical context (Gabbay & Le May, 2011). Trusted colleagues were valued not only for supportive relationships but also for their knowledge, with exchanges facilitating practical know‐how transfer and highlighting the value of *access to relevant expertise* (Gabbay & Le May, 2011).

The *interaction* conditions strongly shaped how peer support was sought and experienced, particularly through the quality of peer relationships. *Hierarchy* operated through both positional authority and experiential differences. When linked to positional authority, such as managerial roles, participants were less likely to perceive that person as a peer, and relational safety was often reduced. Nevertheless, individuals in coordination roles could still enact supportive functions, as noted by Coriasco and colleagues (2025) in their study on the role of nurse navigators. Experiential hierarchy was sometimes perceived as supportive when experienced colleagues were approachable but could also constrain open expression and professional autonomy. Consistent with broader health‐care literature, such hierarchical dynamics influence what is considered acceptable to say, to whom, and under what conditions, with implications for communication, wellbeing, and service delivery (Essex et al., 2023). Importantly, knowledge sharing within peer support relationships was not unidirectional but occurred through mutual contributions. As noted by Murphy (2012), whereas senior practitioners may transmit experiential knowledge, junior practitioners also contribute by sharing up‐to‐date perspectives and emerging practices. Trust and *feeling safe* emerged as foundational conditions for meaningful peer support, enabling vulnerability, openness, and the sharing of uncertainty (Jackson et al., 2023). In their absence, professionals may maintain façades or avoid seeking support (Glenn & Gilbert‐Hunt, 2012). Within such a safe relational climate, *dialogical conditions* were central. Reflective and co‐learning approaches were preferred over directive input, aligning with dialogical learning theories emphasising open‐ended inquiry and collective sense‐making (Houde, 2022; Marshall et al., 2022). However, participants' accounts also suggested that overly reassuring and uncritical dialogue could reinforce habitual practices, suggesting that peer support may hinder learning when insufficiently reflective or disconnected from broader evidence, a dynamic the research team interpreted as echo chambers. Selecting peers with recognised expertise or scholarly orientations may therefore strengthen evidence‐informed reasoning (Hallé et al., 2018).

*Practice context* shaped access to and engagement in peer support. *Workplace culture* characterised by openness, accessibility, and respect facilitated peer dialogue, whereas productivity pressures—especially in private practice—often constrained opportunities for discussion (Welch & Dawson, 2006). Beyond this, collegiality, peer recognition, and openness to change shape engagement in mutual support, as peer cues act as affordances that sustain both competent professional action and the interdependent mobilisation of competence within teams (Thomas, Rochette, et al., 2023). Building on this, the literature highlights the importance of creating open and flexible relational spaces that foster connections, knowledge sharing, and wellbeing, which requires the active involvement of both team members and leaders in shaping a workplace climate conducive to dialogue and collective reflection (Lokhtina & Faller, 2024).

Although spontaneous conversations met immediate needs, *protected time* was viewed as essential for deeper reflection and richer exchanges (Eraut, 2004), as well as for promoting team learning (Nachtergaele et al., 2024). However, disparities between the public and private sectors raised concerns about equity in access to peer support. These differences were closely tied to organisational arrangements: In private practice, peer support was often described as individually negotiated and constrained by back‐to‐back appointments, whereas in public sector contexts, it was more frequently recognised and integrated into organisational routines. These findings align with Eraut's (2004) view of workplace learning as episodic and context dependent, in which time availability influences the cognitive modes practitioners can adopt, ranging from immediate action to deeper reflection. Within this temporal framing, heavy workloads and productivity targets constrain professionals' control over time—a key condition of professional agency—thereby limiting opportunities for sustained peer dialogue and reflective engagement (Thomas, Rochette, et al., 2023).

Importantly, participants' accounts suggest that engaging in peer support was about creating the conditions necessary for reflective engagement with practice rather than about acquiring new factual knowledge. Through dialogical exchanges grounded in shared clinical realities, peer support enabled practitioners to reflexively articulate uncertainty, examine assumptions, and make sense of complex situations collectively. In this way, peer support functioned as a catalyst for experiential learning rather than as a mechanism for knowledge transmission alone.

### 4.3 CPD outcomes of peer support

Outcomes of peer support reflected both immediate and cumulative CPD‐related outcomes rather than a linear sequence. Peer exchanges encouraged participants to question assumptions and reconsider routines, thereby *broadening their perspective*. Similar findings show that peer discussions can challenge practice (Steenbergen & Mackenzie, 2004) and foster self‐awareness (Herkt & Hocking, 2007). Although not explicitly reported by participants, it is possible that peer discussions also served as a way of revisiting critical incidents in practice, thereby supporting self‐awareness and the ability to adopt different perspectives on a situation (Vachon & LeBlanc, 2011).

Peer support also facilitated *risk‐taking and practice‐based experimentation* by providing reassurance and shared reasoning, enabling practitioners to trial new approaches in a clinical context and to manage uncertainty (Eraut, 2004). In this study, ‘risk‐taking’ referred less to high‐stakes practice‐based experimentation than to trialling interventions after discussing or observing them with peers. Beyond encouraging action, peer support appeared to reduce the perceived emotional and professional risk associated with practice change. The literature suggests that shared responsibility, dialogical sense‐making, and collective reflection create conditions that make experimentation more acceptable and less threatening, thereby facilitating the transition from theory to practice (Craven et al., 2021; Houde, 2022). From this perspective, peer support functions not merely as a source of evidence‐based practice but as a relational mechanism that supports practitioners' capacity to engage with uncertainty and enact change in practice.

Validation from peers strengthened participants' confidence in managing future situations, thereby supporting the development of *self‐efficacy*. However, participants' accounts also indicated that directive dialogue could undermine self‐belief, underscoring the importance of feedback literacy in peer exchanges (Carless & Boud, 2018). Although professional identity was not a central theme in participants' narratives, existing literature suggests that peer support processes that enhance self‐efficacy may also contribute, over time, to professional resilience and identity consolidation (Ashby et al., 2013; Jackson et al., 2023). From this perspective, the effects of peer support extend beyond immediate problem‐solving to support longer term professional confidence and engagement.

*Transferring professional knowledge* through peer support reflected participants' desire to share practical wisdom with colleagues and future generations, echoing the concept of *phronesis* as an embodied and ethically grounded form of professional knowledge (Kinsella & Pitman, 2012). According to Gabbay and Le May (2011), such peer‐based exchanges contribute, beyond individual learning, to the development of collective mindlines and the preservation of organisational memory. In this sense, peer support functioned as a mechanism through which experiential knowledge was preserved, circulated, and continually reshaped within teams. The contribution of experienced therapists supported continuity of practice and organisational memory, aligning with the vision of learning organisations in which knowledge is collectively maintained and renewed over time (Garvin et al., 2008). This sharing highlights how peer support enables the transmission of expertise rooted in experience, while renewing practice through dialogue with newer colleagues.

Overall, peer support influenced both individual learning and the collective learning capacity of teams. Within the developed theory, peer support emerged as an immediate, relational, and contextually grounded form of CPD, facilitating reflection, reasoning, and wellbeing. Such exchanges contributed to CPD in ways distinct from formal strategies, cultivating tacit knowledge and practical wisdom, which may complement evidence‐informed practice when embedded in reflective and dialogical processes.

## 5 LIMITATIONS

A strength of this study is its rigorous qualitative analysis process, which enhanced the credibility and trustworthiness of the findings and supported the development of a substantive explanatory theory on the contribution of peer support in occupational therapists' CPD. Several limitations should nevertheless be acknowledged. Recruitment relied on an initial snowball sampling, which may have limited the transferability, as occupational therapists with a similar interest in peer support may have been more likely to participate. In addition, interviews were adapted to the topics prioritised by participants, meaning that some questions from the interview guide were not addressed systematically. As a result, certain themes included in the original guide were not explored in depth, including how peer exchanges explicitly intersected with evidence‐informed practice. Similarly, although most participants both offered and received peer support, their accounts focussed predominantly on the experiences of receiving support, limiting insight into the processes and challenges involved in providing peer support. Future studies may benefit from explicitly examining these reciprocal dynamics. The explanatory theory reflects a context in which peer support was broadly defined and encompassed multiple formal and semi‐structured forms. Future research could refine this theory by more clearly distinguishing informal peer support from formal or organisation‐led support. Finally, both the participants and the research team were situated within a Western health‐care and professional context, which may have shaped how peer support and CPD were understood. Other cultural or epistemological perspectives, including more collective or relational ways of knowing, were not explored and may offer complementary or alternative understandings of peer support.

## AUTHOR CONTRIBUTIONS

Emmanuelle Moreau wrote the article under the supervision of Brigitte Vachon and Annie Rochette. In collaboration with the full team, Emmanuelle Moreau developed the project, including its objectives and methods. Emmanuelle Moreau also conducted data collection and carried out the analysis of the results. The analysis process underwent five iterative rounds with Brigitte Vachon and Annie Rochette. Aliki Thomas and Marie‐Ève Caty reviewed the initial protocol, participated in the final cycle of result analysis, and critically revised the manuscript.

## CONFLICT OF INTEREST STATEMENT

The authors have no conflict of interest to declare.

## DECLARATION OF USE OF ARTIFICIAL INTELLIGENCE

Grammarly was used to enhance the clarity and fluency of Australian English expression and to assist with sentence refinement in this document, as the first author is not a native English speaker. All authors reviewed and approved the final version of the manuscript. One author is more comfortable writing in English than in French, whereas the others, although francophone, are fully bilingual.

## Supporting information


**Data S1.** Supporting Information.

## Data Availability

The data that support the findings of this study are available on request from the corresponding author. The data are not publicly available due to privacy or ethical restrictions.
