# Supplementary material for: Workplace peer support for continuing professional development in occupational therapy: A grounded theory
Source: Aust Occup Ther J. 2026 May 5;73(3):e70091. doi: 10.1111/1440-1630.70091 (PMC13142077; doi:10.1111/1440-1630.70091)
Supplement: Supplementary file 1 — Data S1. Supporting Information. [file AOT-73-0-s001.docx]

**Supplementary file** – Eligibility and socio-demographic online questionnaire

| **Questions** | **Answer options** |
| --- | --- |
| **Eligibility questions** | |
| Are you a member of the Ordre des ergothérapeutes du Québec? | Yes / No / Other (e.g. parental leave) |
| Have you experienced peer support in the past two years? | Yes / No |
| Have you experienced offering peer support in the past two years? | Yes / No |
| What is your practice area? | Public Sector / Private Sector |
| What type of peer support do you mainly use or offer? | Formal (e.g. clinical supervision, community of practice)  Informal (e.g. discussion with a colleague) |
| To what type of peer support do you have access? | Coaching / Co-development sessions / Community of practice / Scheduled discussion with a colleague / Unplanned discussion with a colleague / Mentorship / Sponsorship / Clinical supervision / Clinical and administrative supervision / Other |
| **Socio-demographic questions to describe the participants in this study** | |
| How old are you? | Free space. Numerical value |
| What gender do you identify with? | Female / Male / Neither or both / Prefer not to answer |
| What's your current status? | Single / In couple / In couple with children / Single-parent family / Prefer not to answer / Other |
| What is your last degree obtained? | Bachelor's degree / Master’s degree / Doctorate’s degree / Prefer not to answer |
| What kind of job do you currently hold? If you have more than one job, please refer to the one you consider to be your main one. | Occupational therapist / Planning, programming and research agent / Manager / Other |
| How many years of experience do you have as an occupational therapist? | Free space. Numerical value |
| How many years of experience do you have in your current job in the company? | Free space. Numerical value |
| What is your job status? | Permanent full-time / Temporary full-time / Permanent part-time / Temporary part-time / Under contract |
| Which statement best represents your practice setting? | Rehabilitation center / Long-term care facility / Research center / Hospital Center / Local Community Service Centre / Private clinic / Autonomous practice / School system / University / Other (specify) |
| What is your main area of practice? | Physical health / Mental health / Not applicable |
| With which client(s) do you practice occupational therapy? | Early childhood (0-4 years) / Youth (ages 5-17) / Adults (ages 18-64) / Elderly (65 and over) / Not applicable |
| What type of surroundings do you practice in? | Urban area / Semi-urban area / Rural area / Other (specify) |
| **Section on your workplace** | |
| What is the size of your proximity team? Indicate the number of professionals on your interdisciplinary team, including yourself. | 1 to 5 / 6 to 10 / 11 to 15 / 16 to 20 / Other (specify) |
| How many occupational therapists are on your outreach team? | I'm the only occupational therapist / 2 to 3 / 4 to 5 / 6 or more / Other (specify) |
